# Membrane Clustering of Coronavirus Variants Using Document Similarity

**DOI:** 10.3390/genes13111966

**Published:** 2022-10-28

**Authors:** Péter Lehotay-Kéry, Attila Kiss

**Affiliations:** 1 Department of Information Systems, ELTE Eötvös Loránd University, 1117 Budapest, Hungary; 2 Department of Informatics, J. Selye University, 945 01 Komárno, Slovakia

**Keywords:** document similarity, Doc2Vec, MinHash, genome, bioinformatics, COVID, coronavirus, clustering, P systems, membrane computing

## Abstract

Currently, as an effect of the COVID-19 pandemic, bioinformatics, genomics, and biological computations are gaining increased attention. Genomes of viruses can be represented by character strings based on their nucleobases. Document similarity metrics can be applied to these strings to measure their similarities. Clustering algorithms can be applied to the results of their document similarities to cluster them. P systems or membrane systems are computation models inspired by the flow of information in the membrane cells. These can be used for various purposes, one of them being data clustering. This paper studies a novel and versatile clustering method for genomes and the utilization of such membrane clustering models using document similarity metrics, which is not yet a well-studied use of membrane clustering models.

## 1. Introduction

*Deoxyribonucleic acid* (*DNA*) is a complex molecule consisting of nucleotides. It contains genetic information. Nucleotides consist of three components: nucleobases, a sugar called deoxyribose, and a phosphate group. There are four kinds of nucleobases: adenine, cytosine, guanine, and thymine. The list of the nucleobases of the chromosomes of individuals and species is contained in the genome sequences. These genomes are stored as strings, composed of the first characters of the nucleobases. In the case of a DNA genome, these are ‘A’, ‘C’, ‘G’, and ‘T’ [1].

In this paper, we use text similarity metrics, such as *Doc2Vec* and *MinHash*, to calculate the similarity of virus genomes. Text similarity metrics can be used to assign vectors to texts and then measure their distances in the vector space. The first step in our experiments, was to study how well these metrics can be used to calculate the similarity or difference of genomes. Then with the use of a *membrane clustering* algorithm, we calculated regular and hierarchical clusterings of coronavirus variants. The algorithm first determines centroids in the space, one for each cluster. Then by using some evolutional rules—in our case the *Particle Swarm Optimization* (*PSO*)—we calculated the next configuration in each round until a given number of steps was taken. In the second step of our experiments, we presented the clusters of viral genomes that were created by our method. At the end of the paper, the method was also compared with the usage of *K-Means*. The advantages of using our proposed algorithm are shown by comparing the clustering validity indexes of the clusters produced by our proposed method and by *K-Means*.

The paper is arranged as follows. Section 2 presents some previous studies that are related to this paper, including studies about the usage of document similarity metrics in the comparison of genomes, the clustering of genomes, or membrane computing or membrane-based clustering. In Section 3.1, Section 3.2, Section 3.3 and Section 3.4 the similarity metrics that we used are presented. In Section 3.5 and Section 3.6 the concepts related to clustering algorithms and membrane systems are presented. Then, we present previous studies that we have built upon in this paper in Section 3.7. In Section 4 we present the new methods added to our algorithm that were used to cluster genomes and we also describe how we experimented with these methods.

In more detail, Section 4.1 discusses how we used the similarity metrics to measure the similarity of genomes. Then, Section 4.2 presents our hierarchic *membrane clustering* method, the experiments, and how we connected this method with the genome similarity results. Section 5.1 and Section 5.2 contain the evaluation of using the described similarity metrics on genomes. Then, Section 5.3 and Section 5.4 describe the clustering results we obtained with our *membrane clustering* methods on genomes, including a comparison with the results of another clustering algorithm. The final conclusions are gathered in Section 6. Furthermore, in Section 6, possibilities are suggested for future research.

## 2. Related Works

In this section, we gathered some studies that are related to this paper. First, we present some studies discussing genome similarity metrics. In [2], the authors designed and implemented *SimilarityAtScale*, a communication-efficient distributed algorithm for computing the *Jaccard similarity* among pairs of large datasets. They packaged their routines in a tool called *GenomeAtScale*, which combines the proposed algorithm with tools for processing input sequences.

The authors of [3] introduced the *MinHash Alignment Process* (*MHAP*) for overlapping noisy, long reads using probabilistic, locality-sensitive hashing. In [4], the authors introduced Mash, extending the *MinHash* dimensionality-reduction technique to include a *p* value significance test and a pairwise mutation distance. Their method reduced sequence sets and large sequences to small, representative sketches, from which global mutation distances can be rapidly estimated.

The authors of [5] introduced the containment *MinHash* approach, for estimating the *Jaccard index* of sets of different sizes by leveraging another probabilistic method, Bloom filters for fast membership queries. In [6], the authors introduced *Mashtree*, which uses min-hash values to cluster genomes into trees using the neighbor-joining algorithm.

The authors of [7] proposed an automatic feature learning approach to avoid explicit and predefined feature extraction. The proposed approach is based on the adaptation of two extensively used natural language processing techniques, namely *Word2Vec* and *Doc2Vec*. In [8], the authors applied an unsupervised sequence embedding technique (*Doc2Vec*) to represent protein sequences as rich feature vectors with a low dimensionality. Training a *Random Forest* (*RF*) classifier through a training dataset that covers known *PPIs* (*protein–protein interactions*) between humans and all viruses, they obtained excellent predictive accuracy that outperformed various combinations of machine learning algorithms and commonly-used sequence encoding schemes.

Considering the related studies in the field of coronavirus genome studies, the authors of [9] proposed a method for predicting coronavirus disease 19 (*COVID-19*). They introduced similarity features to distinguish COVID-19 from other human coronaviruses. In [10], the authors created a protocol for the analysis and phylogenetic clustering of *SARS-CoV-2* genomes using an open-source tool, *Nextstrain*, for real-time interactive visualization of genome sequencing data.

Next, we present some related studies that discuss clustering methods. First we discuss genome clustering and then membrane-based clustering. The authors of [1] summarized the biological background of the clustering and classifying of genomes. Then they presented the mathematical models used to analyze documents containing natural languages, such as string distances and the n-gram technique. They also analyzed the language of DNA text. In the end, they used all these technologies to introduce the clustering of genomes. In [11], the authors presented algorithms using nucleotide n-grams that required no preprocessing steps such as sequence alignment—which solved the problems of classification and hierarchical clustering of isolates—to determine the family of genomes and where the given genome belongs. They also introduced a new distance measure between n-gram profiles.

Based on [12], *P systems*, also known as *membrane systems*, a class of distributed parallel computing models was widely used to solve clustering problems. An improved *PSO*-based (*Particle Swarm Optimization*) clustering algorithm inspired by a tissue-like *P system* was introduced in this paper. The proposed clustering algorithm adopted the tissue-like *P system* structure, which contains a loop of cells. A group of candidate cluster centers was represented by an object in the cells. Communication and evolution rules were also adopted in this approach. A local neighborhood topology was built using the communication rules, by virtue of the loop structure of cells. This increased the diversity of objects in the system and promoted the co-evolution of the objects. The different *PSO*-based evolution rules are also used to evolve poor objects and common objects, respectively.

In [13], the authors introduced a variant of a tissue-like P system with active membranes for the clustering process in the calculation of the density of data points using the K-nearest neighbors and Shannon entropy. The authors of [14] proposed an improved spectral clustering algorithm based on a cell-like *P system*. Instead of the *K-Means* algorithm they used the bisecting *K-Means* algorithm. To improve the spectral clustering, as the framework of this algorithm, they constructed a cell-like *P system*. The efficiency of the bisecting *K-Means* is improved by the maximum parallelism of the *P system*.

A summary of the analyzed literature can be seen in Table 1. Many of the related studies mention the variety of their dataset as a limitation of their study. Usually, this means that many of the methods were only validated on given genomes or they were not validated outside the field of computational biology. Some of the related studies also mention the lack of improved database construction. In this paper, we still focus on a given set of genomes as the dataset because our method has previously been validated on other kinds of data outside the field of computational biology in our previous works. These studies also analyzed the utilization of different database management systems as data sources [15,16].

## 3. Materials and Methods

### 3.1. Cosine Similarity

*Cosine similarity* is a similarity measure between two sequences of numbers or vectors. It is the cosine angle between two cardinal vectors of an inner product space. The similarity belongs to the interval of $\left[-1,1\right]$. Proportional vectors have a similarity of 1, orthogonal vectors have a similarity of 0, and opposite vectors have a similarity of −1. They are especially used in a positive space, where the result is between $\left[0,1\right]$. If the vectors are orthogonal, then they are dissimilar and if the unit vectors are parallel, then they are ultimately similar.

This corresponds to the cosine which is null when the components are perpendicular and maximum when the components span a zero angle. Given the vectors A and B, cos(θ), the *Cosine similarity* is represented using a dot product and magnitude, as

(1) $$sim=\cos\left(\theta \right)=\frac{A\cdot B}{\left\|A\|\|B\right\|}.$$

where

(2) $$A\cdot B=\sum_{i=1}^{n}A_{i}B_{i}$$

and

(3) $$\left\|A\|\|B\right\|=\sqrt{\sum_{i=1}^{n}A_{i}^{2}}\sqrt{\sum_{i=1}^{n}B_{i}^{2}}$$

### 3.2. Jaccard Similarity

The dissimilarity and similarity of two sets can be measured with the *Jaccard similarity*, which is usually used to find documents that are textually similar. It is defined by the size of the intersection divided by the size of the union of two sets:

(4) $$J\left(A,B\right)=\frac{\left|A\cap B\right|}{\left|A\cup B\right|}=\frac{\left|A\cap B\right|}{\left|A|+|B|-|A\cap B\right|}$$

where 0≤J(A,B)≤1. If A and B sets are both empty, then their similarity is J(A,B)=1 [17].

### 3.3. MinHash

The similarity of two sets can be calculated rapidly by *MinHash*, as introduced in [18]. It can be applied to large-scale clustering problems, for example, clustering documents based on their similarity of sets of words or detecting duplicate web pages. Similarly, we are going to use it to cluster genomes based on the similarity of their sets of n-grams.

Let *A* and *B* be two subsets of set *U*. The minimal member of any set *S* with regard to h∘perm (the member *x* of *S* with the minimal value of h(perm(x)) is defined as $h_{min}\left(S\right)$, where perm is a random permutation of the elements of *U* and *h* is a hash function mapping the members of *U* to individual numbers. Applying $h_{min}$ on *A* and *B* both assuming there is no hash conflict, $h_{min}\left(A\right)$ and $h_{min}\left(B\right)$ values are equal if and only if among all elements of A∪B, the A∩B intersection contains the minimum hash value element. The probability of this case is:

(5) $$Pr[h_{min}\left(A\right)=h_{min}\left(B\right)]=J\left(A,B\right)$$

### 3.4. Word2Vec and Doc2Vec

An open-source tool was introduced in [19] for natural language processing that is called *Word2Vec*. It proposes effective algorithms to represent words as word embeddings that are N-dimensional real vectors. It is also used to measure the similarity of words. The algorithm uses a simple neural network model with a single hidden layer. Taking a large text as input, the algorithm assigns a vector for each distinct word, creating a vector space consisting of hundreds of dimensions. Similar context words are located close to each other in the vector space. These vectors are selected cautiously so that the mathematical function *Cosine similarity* can state the semantic similarity between the words that are represented by vectors. It has two models, *continuous bag-of-words* (*CBOW*) and *Skip–Gram*.

*Doc2Vec* is an extension of *Word2vec* that was proposed in [20]. It constructs embeddings regardless of documents of any lengths. It also uses a vector called *Paragraph ID* (or doc ID). Two algorithms are used to calculate *Doc2Vec*. One is similar to the *CBOW* model, called *Distributed Memory version of Paragraph Vector* (*PV-DM*). The other one is called *Distributed Bag of Words version of Paragraph Vector* (*PV-DBOW*), which is similar to *Skip-Gram*.

In this paper, we used *Skip-Gram*, which converts each word into a feature vector using *Word2Vec*. Then it calculates the average of these vectors, resulting in the *Doc2Vec* vectors. Their length is the same as the length of *Word2Vec* vectors. Formally:

(6) $$D2V=\frac{W2V\left(w_{1}\right)+\cdots +W2V\left(w_{n}\right)}{n}$$

where $W2V(w_{i})$ represents the $i^{th}$ word’s W2V vector and *n* is the number of vectors.

### 3.5. Clustering Algorithms

The objective of clustering algorithms is, by using a function of goodness, to discover groupings of a specified data set, where each data point of the set belongs to a group. In such a cluster, the similarity—in our case document similarity—of the members is maximized, while in separate groups, the similarity of the points is as low as possible. To solve this clustering problem, many distinct approaches exist (centroid-based, density-based, connectivity-based), each bearing its strong points.

At the end of the paper, we are going to compare our clustering algorithm with *K-Means* [21]. This algorithm partitions a set of *n* data vectors $x_{0},\ldots ,x_{n}\in X$ into *k* disjoint clusters that are described by the mean $c_{0},\ldots ,c_{k}\in C$ of the samples contained in the cluster. The algorithm clusters the samples by minimizing the sum of squares in each cluster:

(7) $$\sum_{i=0}^{n}\min_{c_{j}\in C}\left(|\right|x_{i}-c_{j}{\left|\right|}^{2})$$

The goodness of clustering can be measured by multiple methods. In this paper, we are going to use the *Davies–Bouldin index* [22] and the Silhouette coefficient [23] to compare the results of our membrane-based clustering method with the results of *K-Means*.

The *Davies–Bouldin index* is the average similarity of each cluster with its most similar cluster. This similarity is the ratio of the distances of the points within the cluster and the distances of the points between the clusters. This means that clusters that are less dispersed and further apart result in a better score.

The *Silhouette coefficient* is measured using two values: for each sample, *a* is the mean intra-cluster distance and *b* is the mean nearest cluster distance. With these values, the coefficient is calculated in the following way:

(8) (b−a)/max(a,b)

### 3.6. Membrane Computing

Various types of membranes delimit the parts in a *membrane system* or *P system*. Membranes keep together certain chemicals and allow other chemicals to pass selectively in biology and chemistry. These systems take the form of a certain structure. A cell-like membrane system takes the form of a tree, while a tissue-like membrane system takes the form of an arbitrary graph. These structures consist of parallel computing units called cells.

Evolution rules are contained in the regions defined by these cells. These rules delineate the calculations in the system as a sequence of transitions between the states of the system. A multiset of objects is also contained in a region. When the system takes a step in the computation, it chooses non-deterministically, in a maximally parallel manner from all the available rules.

The system reaches a new state or configuration when a step is applied. Furthermore, when there is no possibility for any transitions, meaning there are no rules in any of the cells that could be applied, the calculation terminates. After the system halts, the result of the computation may be defined by the state of a specific cell [24].

In the case of the clustering task, an object is going to be a vector containing the potential cluster centroids. The evolution rules are going to move these centroids of the objects of each cell in each step until a given number of steps. The centroids start from an initial state randomly chosen from the data points to be clustered.

### 3.7. Our Approach from Our Previous Works

We partially used the same *membrane clustering* method that we used in two of our previous studies [15,16]. In the first study, we were experimenting with membrane-based clustering on data points stored in *PostgreSQL DBMS*. We also validated our clustering using the *Davies–Bouldin index*, the Silhouette coefficient, and the *Calinski–Harabasz index*. In this current paper, we are going to use the input parameters as we have found them to be optimally set in this previous work.

In our second previous study, we performed some experiments using greater datasets stored in *NoSQL DBMSs*: *Redis* and *MongoDB*. We evaluated the running time, storage size, and memory usage of these systems in combination with our algorithm. The detailed description of the algorithm and equations that we used in this study can also be found in that paper.

## 4. Experiments and the Algorithm

In this paper, we added the document similarity underneath our algorithm and a hierarchical system above it. In this section, we walk through these extensions and explain our experiments.

### 4.1. Experiments on Using Doc2Vec and MinHash

In the first two experiments, we performed simple tests where we calculated the distance of the *Doc2Vec* and *MinHash* vectors of three viruses: *Human Coronavirus 229E*, *Human Coronavirus NL63*, and *Hepatitis C*. We wanted to create associations between smaller parts of the text. In order to do this, we wanted to split these lines into smaller parts of size *m*. We will call these elements *m-grams* from now on.

Next, we trained the model. For this, we simply used every genome we had on every *m* size we wanted to analyze. We used the trained model to calculate the vectors for two genomes and then used *Cosine distance* to obtain their similarity. *Euclidean distance* could also be used and would have produced roughly the same results.

For *MinHash*, we used the same examples that we tried in the previous model with *Jaccard similarity*.

### 4.2. Hierarchic Membrane Clustering

In the following section, we describe our hierarchic *membrane clustering* method and the four experiments we created to evaluate it using *Doc2Vec* and *MinHash*. The following elements are going to be used in the pseudo codes:

- *c*—a cluster containing a list of vectors.
- *t*—threshold, the size of clusters we want to create.
- memb_clust—our original *membrane clustering* algorithm described in detail in [16].
- docs—a list of documents or, in our case, genomes.
- *m*—the size of the *m-grams*.
- doc2vec_model—creates the *Doc2Vec* model described in [20] and in Section 3.4.
- read_genome—reads the *m-grams* of a document or genome of size *m*.
- create_vect—creates the vector of a genome based on the model.
- cos_dist—the distance of two vectors, as described in Section 3.1.
- normalize—normalizes a vector.

Hierarchical clustering differs from regular clustering in that a cluster can become part of another cluster. Our *hierarchical membrane clustering* recursively calls our original *membrane clustering* method, splitting a greater clustering result *c* of the previous clustering round into two (or *N*) branches until all clusters contain only a maximal number of samples, which is smaller than the *t* threshold.

Pseudo code for hierarchical membrane clustering:


FUNCTION hierarchic_memb_clust(c, t)
 result = memb_clust(c)
 FOR each c IN result DO
  IF size(c) > t
   hierarchic_memb_clust(c)
END


For example, in the experiments using hierarchical clustering, we created clusters containing two elements and divided the greater clusters into two parts in each round.

As it can be seen in Section 5, we were experimenting with the usage of document similarity metrics with our clustering in four ways.

Our first idea was to represent each virus as a vector with *Doc2Vec* and run the clustering algorithm on the results. To achieve this we first needed to be able to obtain the vector for just one genome. Then we applied this algorithm to all genomes docs and stored their vectors in a list. Here, we used a fixed *m* for the *m-grams*.

Pseudo code for the first experiment:


FUNCTION clust_doc2vec(docs, m)
 model = doc2vec_model(docs)
 FOR each doc IN docs DO
  genome = read_genome(doc, m)
  vect = create_vect(model, genome)
  ADD vect TO vectors
 hierarchic_memb_clust(vectors)
END


**Figure 1:**
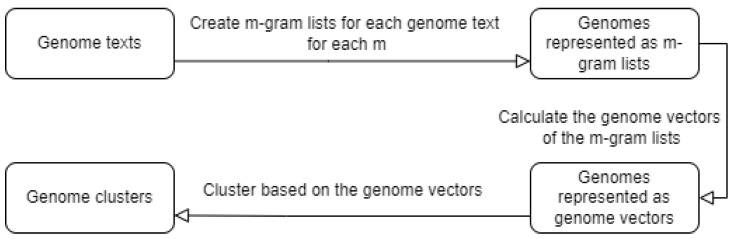
Workflow of the second experiment.

For our second experiment, we did the same thing, but instead of selecting a specific *m m-gram* size, we concatenated the vectors for each size, thus trying to obtain as much stored information as possible. A workflow of this second experiment can be seen in Figure 1. The workflow for the first experiment would be similar, but with only creating one *m-gram* list for each genome.

Pseudo code for the second experiment:


FUNCTION clust_doc2vec(docs)
 model = doc2vec_model(docs)
 FOR each doc IN docs DO
  FOR each m DO
   genome = read_genome(doc, m)
   vector = create_vect(model, genome)
   ADD vect TO vector_of_genome
  ADD vector_of_genome TO vectors
 hierarchic_memb_clust(vectors)
END


In the next two experiments, we applied the tested metrics to all pairs of genomes with fixed *m*. So the final output for each genome is a vector containing its similarity to each other genome. First, we tried this with *Doc2Vec*.

Pseudo code for the third experiment:


FUNCTION clust_cos_dist(docs, m)
 model = doc2vec_model(docs)
 FOR each d1 IN documents DO
  FOR each d2 IN documents DO
   g1 = read_genome(d1, m)
   g2 = read_genome(d2, m)
   v1 = create_vect(model, genome1)
   v2 = create_vect(model, genome2)
   dist = cos_dist(v1, v2)
   norm_dist = normalize(dist)
   ADD norm_dist TO distances
  ADD distances TO distances_list
 hierarchic_memb_clust(distances_list)
END


**Figure 2:**
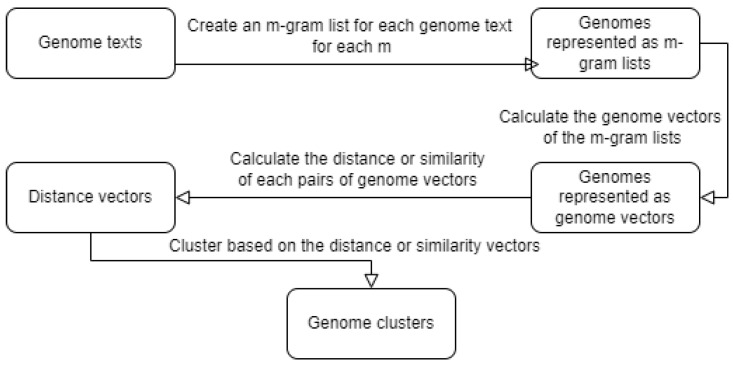
Workflow of the third and fourth experiments.

In the last experiment for our hierarchic clustering, we used the same methodology but with *MinHash* and *Jaccard similarity*. A workflow of this third and the last experiments can be seen in Figure 2.

## 5. Results

In this section, we present the results that we obtained with the methods described in Section 4. We used the *NCBI reference sequences* (*RefSeq*) [25] and *The European nucleotide archive* [26] databases to collect the genomes used in the following.

### 5.1. Evaluation of Using Doc2Vec

We examined the ways text similarity metrics can be used to examine the similarity of genomes, with most attention focused on the viruses described in Section 4.1. For *Doc2Vec*, we used *Gensim*’s implementation [27].

In Table 2, we calculated the distance of *Human Coronavirus 229E–Hepatitis C* (denoted as *C1*), *Human Coronavirus NL63–Hepatitis C* (denoted as *C2*), and *Human Coronavirus 229E–Human Coronavirus NL63* (denoted as *C3*). The metric goes from 0 to 1 and the smaller number means that the two genomes are more similar. We wanted to determine the ideal *m-gram* size. The distance between the two *Corona* variants must be smaller than the distance between *Corona* and *Hepatitis C* virus. So we calculated the difference between the distances too: in column *C1–C3* the difference between the first and third column and in column *C2–C3* the difference between the second and the third column. In Table 3, we calculated the running times for the calculations of the above and the average in seconds for each *m*.

Based on the test results that can be seen in Table 2 we found that the ideal *m* is 14 in this case since there we can see the two *Corona* variants to be the closest to each other compared to the *Hepatitis C* virus. Usually based on other experiments, for other data, *m* between 3 and 14 gives valid results. Below that the genomes are too uniform, above that there are too many unique sequences, and the similarity between genomes is too close to 0.

Considering only the scores, m=14 seems to be the best. If we also consider the running times in Table 2, we can also see that with the increasing *m*, the running time decreases. In this experiment with m=14, our score and running time are also fine. However, if under other circumstances, such as using other data, a smaller *m* is found to be optimal; therefore, we can conclude that it is possible to increase the *m* for a smaller running time, if needed, until 14, since the scores are usually still valid between the range of 3 and 14.

### 5.2. Evaluation of Using MinHash

To experiment with *MinHash*, we used the implementation of datasketch [28].

The results can be seen in Table 4. The metric still goes from 0 to 1, but the higher it is the more similar the two genomes are. Based on this experiment, *m* between 5 and 8 provided the correct results, because it measured a bigger similarity between the two *Corona* variants compared to the similarity of the *Hepatitis C* and a *Corona* variant. However, when m<5 and when m>8, the results seem incorrect in most cases. With these *m* values, sometimes greater or equal similarity was measured between the *Hepatitis C* and a *Corona* variant than between the two *Corona* variants. Furthermore, when m>8, it started to measure 0 distances between the different genome sequences.

We measured the running times again in Table 5. However, when m<2 and when m>10 *Doc2Vec* is faster, we would not use these values anyway considering the distance scores. Overall the running times are smaller for *m* between 5 and 8 in the case of *MinHash*, where we received the best scores. However, these running times are bigger than the running time with m=14 when using *Doc2Vec*, which we would use as a value giving valid results. Furthermore, when m>10, the running times with *Doc2Vec* are smaller.

### 5.3. Creating Clusters Using Hierarchic Membrane Clustering

The next thing we wanted to do is to find a way to efficiently sort multiple viruses into clusters. We used the methods described in Section 4.2. Here this first experiment did not bring satisfying results, so we have omitted the results from this paper.

Our second experiment could effectively distinguish the *murine hepatitis* variants from other viruses. It could also collect *bovine coronavirus* variants into the same cluster, with the exception of one variant. It could collect the *influenza* variants also in the same cluster. It successfully distinguished *SARS CUHK* variants from other variants and viruses. Furthermore, in the end, it collected many other *SARS* variants in the same clusters. It could also collect *SARS Sin* variants into the same cluster correctly. Furthermore, it also nearly clustered the *West Nile* variants correctly, but in the end was unsuccessful.

Sample clusters:


[[’Duck adenovirus’, ’Human Coronavirus NL63’],
[’Murine hepatitis MHV-A59 C12’, ’Murine hepatitis ML-10’],
[’Murine hepatitis 2’, ’Murine hepatitis Penn97-1’]]
 
[[’Bovine coronavirus BCoV-ENT’, ’Bovine coronavirus Mebus’],
[’Bovine coronavirus Quebec’]]
 
[[’Bovine coronavirus BCoV-LUN’, ’Cowpox’, ’Vaccinia’], [’Variola’]]
 
[[’InfluenzaA’, ’InfluenzaB’], [’RotaVirus’]]
 
[’SARS CUHK-Su10’, ’SARS CUHK-W1’]]
 
[[’Galveston’, ’Onyong-nyong’, ’Ross River’, ’SARS civet007’, ’SARS ZJ01’,
’WestNile1’], [’Hepatitis C genotype 1’, ’SARS civet010’, ’WestNile2’]]
 
[[’SARS HKU-39849’, ’SARS Tor2’, ’SARS TW1’, ’SARS Urbani’],
[’SARS Sin2500’, ’SARS Sin2677’, ’SARS Sin2679’, ’SARS Sin2748’,
’SARS Sin2774’]]


For the third experiment, after running some tests, we obtained a good clustering of the viruses using m=11 in our membrane-based hierarchical clustering. In given moments it could separate influenza and *West Nile* variants from the others. It could collect the *SARS Sin* variants together, except for one variant. It collected *murine hepatitis* variants in one cluster, except one variant. Furthermore, it collected the *bovine coronavirus* variants into one cluster, except one. It also clustered *SARS CUHK* variants correctly.

Sample clusters:


[[’Avian infectious brochitis’, ’Human coronavirus 229E’, ’SARS ZJ01’,
’Turkey coronavirus MG10’], [’Variola’]],
 
[[’Bovine coronavirus BCoV-ENT’, ’Bovine coronavirus Mebus’,
’Human Coronavirus HKU1’], [’Bovine coronavirus Quebec’,
’Human Coronavirus NL63’]],
 
[[’Duck adenovirus’, ’SARS civet010’, ’SARS HKU-39849’, ’SARS Tor2’],
[’SARS Sin2500’, ’SARS Sin2677’, ’SARS Sin2679’, ’SARS Sin2774’]],
 
[’SARS TW1’, ’SARS Urbani’],
 
[[’Murine hepatitis 2’, ’Murine hepatitis Penn97-1’, ’Porcine diarrhea’],
[’Murine hepatitis MHV-A59 C12’]],
 
[[’Murine hepatitis ML-10’, ’SARS civet007’], [’SARS Sin2748’]],
 
[[’SARS BJ01’], [’SARS CUHK-Su10’, ’SARS CUHK-W1’]],
 
[’Bovine coronavirus BCoV-LUN’],
 
[’Cowpox’, ’Vaccinia’],
 
[’WestNile1’, ’WestNile2’],
 
[’Ross River’],
 
[’Galveston’, ’Hepatitis C genotype 1’],
 
[’Onyong-nyong’],
 
[’InfluenzaA’, ’InfluenzaB’],
 
[’RotaVirus’]


Here, in the last experiment we used the same methodology, but with *MinHash* set to m=8. It collected *SARS Sin* variants and other *SARS* variants into the same cluster. It could also distinguish between *murine hepatitis* variants and distinguish the *SARS CUHK* variants into the same clusters. However, it was not successful with the other viruses:

Sample clusters:


[[’Galveston’, ’Human coronavirus 229E’, ’SARS BJ01’],
[’Murine hepatitis MHV-A59 C12’, ’SARS ZJ01’, ’WestNile2’]],
 
[[’Hepatitis C genotype 1’, ’Onyong-nyong’, ’SARS civet007’],
[’SARS CUHK-Su10’, ’SARS CUHK-W1’]],
 
[[’Murine hepatitis 2’, ’Murine hepatitis ML-10’,  
’Murine hepatitis Penn97-1’],
[’WestNile1’]],
 
[[’SARS civet010’, ’SARS Sin2500’, ’SARS Sin2677’, ’SARS Sin2679’,
’SARS Sin2748’, ’SARS Sin2774’],
[’SARS HKU-39849’, ’SARS Tor2’, ’SARS TW1’, ’SARS Urbani’]]


Overall, the first and fourth attempts with the similarity matrices produced the worst results; most clusters were essentially random. We did not obtain a satisfying result with *MinHash* in the end, so in the following, we used *Doc2Vec*.

The results of the second and the third *Doc2Vec* tests with correct *m* parameters were mostly correct with one or two viruses out of order. We continued with these parameters in the last tests.

### 5.4. Comparison of our Membrane Based Approach with K-Means

We evaluated the performance of the clustering results based on two clustering validity indices: the *Davies–Bouldin index* and the *Silhouette coefficient* and compared our results with the results received with *K-Means*. We used the implementation of *sklearn* [29]. First, we presented what indexes we reached with our membrane-based clustering and *K-Means* on the results of the third *Doc2Vec* test with m=11.

We present the best *Daves–Bouldin indexes* that we obtained for the different number of clusters with the membrane configuration that we used to reach the scores, compared to the indexes we reached with *K-Means* in Table 6. In the case of the *Daves–Bouldin index*, a lower value means better clustering. It can be seen that we found at least one configuration for each number of clusters where we could reach a better index than the index produced by *K-Means*. The average index reached with our method is also much better. Based on these results, with the increasing number of clusters, the clustering became better and better. Our method with 14 clusters produced the best results.

In Table 7, the best Silhouette indexes can be seen that we reached, similarly to Table 6. In the case of the Silhouette index, a higher value means better clustering. It can be seen that we found at least one configuration for the most number of clusters where we could reach a better index than the index produced by *K-Means*, but not for all number of clusters. The average value reached with our method is also not as much better as with the Davies–Bouldin index. Based on these results, with decreasing number of clusters, the clustering became better and better. Our method with six clusters produced the best result.

Similar to the clustering of the results of the third *Doc2Vec* test with m=11, we will now discuss which indexes we obtained in the second *Doc2Vec* test with *m* set between 4 and 16.

First, we present the best *Daves–Bouldin indexes* that we reached for the different number of clusters with the membrane configuration that we used to reach the scores, compared to the indexes we reached with *K-Means* in Table 8. It can be seen that we found at least one configuration for the most number of clusters where we could reach a better index than the index produced by *K-Means*. Compared to the previous experiment, this experiment showed that not increasing the number of clusters led to better clustering. Moreover, *K-Means* with 9–10 clusters found very good clusterings, with 9 clusters being the best in this experiment, while our algorithm performed the worst with these cluster numbers. Furthermore, it can be seen that the resulting values and averages are better than the resulting values of the previous experiment with a fixed *m*.

We present the best *Silhouette* scores that we obtained for the different number of clusters with the membrane configuration that we used to reach the scores, compared to the indexes we reached with *K-Means*, in Table 9. It can be seen that we found at least one configuration for the most number of clusters where we could reach a better index than the index produced by *K-Means*, but not for all number of clusters. Furthermore, although we produced better values with more cluster numbers, the average with *K-Means* was still better. Again, the best clusterings resulted in nine clusters both with our method and the *K-Means*. Moreover, our method with nine clustersled to the best clustering in this experiment. Furthermore, it can be seen that the resulting values here are better than the resulting values of the previous experiment with a fixed *m*.

## 6. Discussion

### 6.1. Conclusions

We examined various methods and algorithms in the process of clustering genomes. First, we evaluated the usage of *Doc2Vec* and *MinHash* and how they can be used to measure the distance between genomes. After that, we applied our hierarchical *membrane clustering* method to the results of the similarity metrics and validated the results using virus genomes. Then, we compared our *membrane clustering* method with *K-Means* and evaluated their clustering results using the *Silhouette coefficient* and the *Davies–Bouldin index*.

In the end, we can conclude that our membrane clustering method can be effectively used to cluster virus genomes because it reached good scores compared to *K-Means*. We also found that our clustering methods worked better with *Doc2Vec* than with *MinHash*.

### 6.2. Limitations

To achieve better results compared to rival solutions, as a basis for clustering algorithms, evolutionary optimization methods were utilized, including *Particle Swarm Optimization* (*PSO*), *Red Fox Optimization* (*RFO*), *Polar Bear Optimization* (*PBO*), *Chimp Optimization Algorithm* (*ChOA*), or even a combination of these as their frameworks [30,31,32,33]. Making further experiments and evaluations comparing these heuristics was not in the scope of this paper, so we used *PSO*, which we already had built into our *membrane clustering* approach in our previous studies and are described in Section 3.7. However, in future studies, this aspect could also be evaluated in more detail.

We evaluated the clustering performance of our method, but it could still be examined and improved in other aspects. For example, since *membrane systems* are parallel models, the running time of our algorithm could be improved in a distributed or multi-threaded environment.

It would be also interesting to examine and test the usage of classification models to classify genomes such as *Decision Trees*, *Support Vector Machines*, *Random Forests*, *or Neural Networks*.

Furthermore the accuracy of the algorithm could also be tested on larger data sets, for example using other kinds of coronaviruses such as *MERS* [34] and *SARS-CoV* [35]. It would also be interesting to try our algorithm and these models not only on virus genomes but on bacteria or other species to inspect how these longer genome sequences affect the running time, memory usage, etc.

## Figures and Tables

**Table 1:** Literature summary table.

Author	Title	Finding
Besta (2020)	Communication-efficient jaccard similarity for high-performance distributed genome comparisons	*SimilarityAtScale* computes the *Jaccard similarity* among pairs of large datasets. *GenomeAtScale* combines this with sequence processing.
Berlin (2015)	Assembling large genomes with single-molecule sequencing and locality-sensitive hashing	*MHAP* for overlapping reads.
Ondov (2016)	Mash: fast genome and metagenome distance estimation using MinHash	Mash for *MinHash* dimensionality-reduction, to reduce sequence sets and large sequences to small, representative sketches.
Koslicki (2019)	Improving MinHash via the containment index with applications to metagenomic analysis	Containment *MinHash* estimating *Jaccard index* using *Bloom filters*.
Katz (2019)	Mashtree: a rapid comparison of whole genome sequence files	*Mashtree* using *MinHash* to cluster genomes into trees.
Oubounyt (2018)	Deep learning models based on distributed feature representations for alternative splicing prediction	Feature learning, avoiding explicit and predefined feature extraction.
Yang (2020)	Prediction of human–virus and protein–protein interactions through a sequence embedding-based machine learning method	Representing protein sequences as *Doc2Vec* vectors and training *RF* covering *PPIs* between humans and viruses.
Arslan (2021)	COVID-19 prediction based on genome similarity of human SARS-CoV-2 and bat SARS-CoV-like coronavirus	Introducing similarity features to distinguish *COVID-19* from other human *coronaviruses*.
Jolly (2021)	Computational analysis and phylogenetic clustering of SARS-CoV-2 genomes	A protocol for the analysis and clustering of *SARS-COV-2* genomes.
Tomović (2006)	n-Gram-based classification and unsupervised hierarchical clustering of genome sequences	New distance measure between n-gram profiles.
Gao (2018)	An improved PSO-based clustering algorithm inspired by tissue-like P system	Using a local neighborhood topology, increasing the diversity and co-evolution of objects in the *P system*.
Jiang (2019)	A density peak clustering algorithm based on the K-nearest Shannon entropy and tissue-like P system	A *P system* variant for the calculation of density points using the *K-nearest neighbors* and *Shannon entropy*.
Zhang (2019)	An improved spectral clustering algorithm based on cell-like P system	Improved efficiency of *K-Means* in spectral clustering using the maximum parallelism of the *P system*.

**Table 2:** Distance scores (229E-Hepa (*C1*), NL63-Hepa (*C2*), and NL63-229 (*C3*)) of the *Doc2Vec* vectors of the three viruses and the differences of these distances (*C1–C3* and *C2–C3*) using different *m-gram* sizes.

m-Gram Size	229E-Hepa (C1)	NL63-Hepa (C2)	NL63-229 (C3)	C1–C3	C2–C3
1	0.0002123	0.0004236	0.0000394	0.0001729	0.0003842
2	0.3399463	0.3453034	0.0001016	0.3398448	0.3452018
3	0.3695712	0.4787790	0.0276033	0.3419679	0.4511757
4	0.0587197	0.0909740	0.0077233	0.0509964	0.0832507
5	0.0191701	0.0352157	0.0032254	0.0159447	0.0319903
6	0.0110183	0.0153024	0.0034113	0.0076070	0.0118911
7	0.0123603	0.0196021	0.0015012	0.0108591	0.0181009
8	0.0460361	0.0572509	0.0020966	0.0439395	0.0551543
9	0.1026353	0.0958337	0.0105982	0.0920371	0.0852355
10	0.0347726	0.0402217	0.0138344	0.0209382	0.0263873
11	0.0728078	0.0809851	0.0123689	0.0604389	0.0686163
12	0.0049008	0.0044417	0.0007218	0.0041789	0.0037199
13	0.0048270	0.0083499	0.0008653	0.0039617	0.0074846
14	1.0547185	0.8462670	0.2651407	0.7895779	0.5811264
15	0.0053818	0.0038288	0.0004694	0.0049124	0.0033594
16	0.0023146	0.0034332	0.0001939	0.0021207	0.0032393

**Table 3:** Running times at the calculation of the distance scores (229E-Hepa (*C1*), NL63-Hepa (*C2*), and NL63-229 (*C3*)) of the *Doc2Vec* vectors of the three viruses and the average of these running times in seconds using different *m-gram* sizes.

m-Gram Size	229E-Hepa (C1)	NL63-Hepa (C2)	NL63-229 (C3)	Average
1	0.8970317	0.3545133	0.3972148	0.5495866
2	0.4256047	0.4640406	0.4402194	0.4432882
3	0.1884928	0.1884540	0.2870301	0.2213256
4	0.1834050	0.5818737	0.3334340	0.3662376
5	0.1832464	0.2470372	0.2526913	0.2276583
6	0.1697437	0.1836954	0.2287966	0.1940786
7	0.1392266	0.1728073	0.2169918	0.1763419
8	0.1332747	0.1487379	0.2123267	0.1647798
9	0.1160918	0.1238680	0.2051878	0.1483825
10	0.0983900	0.0994981	0.1786392	0.1255091
11	0.0305363	0.0213676	0.0325485	0.0281508
12	0.0218683	0.0311976	0.0345565	0.0292075
13	0.0247284	0.0213287	0.0489603	0.0316725
14	0.0102822	0.0124120	0.0124389	0.0117110
15	0.0299261	0.0334483	0.0347533	0.0327092
16	0.0193578	0.0229860	0.0361347	0.0261595

**Table 4:** Similarity scores (229E-Hepa (*C1*), NL63-Hepa (*C2*), and NL63-229 (*C3*)) of the *MinHash* of the three viruses and the differences of these distances (*C1–C3* and *C2–C3*) using different m-gram sizes.

m-Gram Size	229E-Hepa (C1)	NL63-Hepa (C2)	NL63-229 (C3)	C3–C1	C3–C2
1	1.0000000	1.0000000	1.0000000	0.0000000	0.0000000
2	0.9375000	0.9062500	0.8437500	−0.0937500	−0.0625000
3	0.9687500	0.9609375	0.9921875	0.0234375	0.0312500
4	0.9921875	0.9921875	0.9843750	−0.0078125	−0.0078125
5	0.6406250	0.6640625	0.8437500	0.2031250	0.1796875
6	0.1875000	0.2265625	0.5000000	0.3125000	0.2734375
7	0.0390625	0.0390625	0.2421875	0.2031250	0.2031250
8	0.0078125	0.0156250	0.0625000	0.0546875	0.0468750
9	0.0078125	0.0000000	0.0000000	−0.0078125	0.0000000
10	0.0000000	0.0000000	0.0156250	0.0156250	0.0156250
11	0.0156250	0.0234375	0.0390625	0.0234375	0.0156250
12	0.0000000	0.0000000	0.0000000	0.0000000	0.0000000
13	0.0078125	0.0078125	0.0312500	0.0234375	0.0234375
14	0.0000000	0.0000000	0.0000000	0.0000000	0.0000000
15	0.0000000	0.0000000	0.0000000	0.0000000	0.0000000
16	0.0078125	0.0000000	0.0312500	0.0234375	0.0312500

**Table 5:** Running times at the calculation of the similarity scores (229E-Hepa (*C1*), NL63-Hepa (*C2*), and NL63-229 (*C3*)) of the *MinHash* of the three viruses and the average of these running times in seconds using different *m-gram* sizes.

m-Gram Size	229E-Hepa (C1)	NL63-Hepa (C2)	NL63-229 (C3)	Average
1	0.8962874	0.7013130	0.6866167	0.7614057
2	0.3641375	0.2303220	0.3430197	0.3124931
3	0.2314856	0.1512294	0.1601725	0.1809625
4	0.2043768	0.1776504	0.1927966	0.1916079
5	0.1548510	0.0950320	0.0955275	0.1151368
6	0.1273663	0.0809898	0.0784282	0.0955948
7	0.0987748	0.0653925	0.0646084	0.0762586
8	0.0836217	0.0639499	0.0647426	0.0707714
9	0.0778825	0.0552873	0.0717265	0.0682988
10	0.1202149	0.0506465	0.0780083	0.0829566
11	0.1021783	0.0861990	0.0821483	0.0901752
12	0.1009907	0.0745528	0.0727619	0.0827685
13	0.0592558	0.0629678	0.0499611	0.0573949
14	0.0672873	0.0362306	0.0339905	0.0458361
15	0.0513617	0.0357769	0.0331476	0.0400954
16	0.0549223	0.0350066	0.0339571	0.0412953

**Table 6:** *Davies–Bouldin* scores of the *K-Means* and the *Membrane clustering* with the different number of clusters and *m-gram* size of 11 and using different membrane configurations. The Better scores are bold in the table.

Clusters	Steps	Cells	Objects	Membrane	K-Means
6	7	11	8	**0.8324**	1.4146
7	3	10	9	**0.9717**	1.2747
8	2	11	9	**0.8068**	1.2404
9	3	11	6	**0.9664**	1.0890
10	2	11	8	**0.8110**	1.2967
11	2	10	9	**0.7900**	1.3230
12	2	10	8	**0.8159**	1.0650
13	5	11	6	**0.8198**	1.0837
14	7	11	7	**0.7652**	0.9753
15	4	8	6	**0.8162**	0.9583
16	5	10	7	**0.7743**	0.9807
average				**0.83**	1.15

**Table 7:** *Silhouette* scores of the *K-Means* and the *membrane clustering* with the different number of clusters and *m-gram* size of 11 and using different membrane configurations. The Better scores are bold in the table.

Clusters	Steps	Cells	Objects	Membrane	K-Means
6	8	10	6	**0.3437**	0.3431
7	8	11	7	**0.3411**	0.3304
8	4	8	8	**0.3219**	0.3161
9	3	11	6	**0.3235**	0.3172
10	5	11	6	**0.3256**	0.3236
11	5	11	6	0.2892	**0.3031**
12	6	9	8	**0.3011**	0.2945
13	7	8	7	**0.3083**	0.2499
14	4	8	8	**0.2508**	0.2503
15	4	8	6	**0.2695**	0.2359
16	3	9	6	**0.2384**	0.2378
average				**0.3011**	0.2910

**Table 8:** *Davies–Bouldin* scores of the *K-Means* and the *membrane clustering* with the different number of clusters and m between 4 and 16 and using different membrane configurations. The Better scores are bold in the table.

Clusters	Steps	Cells	Objects	Membrane	K-Means
6	6	11	8	**0.8471**	0.9975
7	4	8	8	**0.8413**	0.8618
8	2	11	9	**0.7800**	0.8202
9	3	7	6	0.9402	**0.7239**
10	5	9	5	0.8748	**0.6684**
11	4	8	8	**0.7226**	0.9235
12	7	11	7	**0.8516**	0.8887
13	5	11	7	**0.7441**	0.8155
14	3	8	6	**0.7625**	0.8215
15	3	8	6	**0.7499**	0.7617
16	7	11	7	**0.7254**	0.7727
average				**0.8035**	0.8232

**Table 9:** *Silhouette* scores of the *K-Means* and the *membrane clustering* with the different number of clusters and m between 4 and 16 and using different membrane configurations. The Better scores are bold in the table.

Clusters	Steps	Cells	Objects	Membrane	K-Means
6	6	11	8	**0.4079**	0.4069
7	5	7	8	0.4396	**0.4452**
8	4	7	8	**0.4483**	0.4385
9	5	8	9	**0.4513**	0.4437
10	5	9	5	0.4130	**0.4467**
11	8	9	6	**0.3916**	0.3839
12	9	10	7	**0.3932**	0.3894
13	7	10	8	**0.3821**	0.3802
14	4	7	8	**0.3832**	0.3745
15	2	11	6	**0.3819**	0.3733
16	7	8	5	0.3473	**0.3707**
average				0.4035	**0.4048**

## Data Availability

We used the NCBI reference sequences (RefSeq) [25] and The European Nucleotide Archive [26] databases to collect the genomes used in this paper.

## Abbreviations

The following abbreviations are used in this manuscript:

COVID19	Coronavirus Disease 19
DNA	Deoxyribonucleic acid
MHAP	MinHash Alignment Process
RF	Random Forest
PPIs	protein–protein interactions
CBOW	continuous bag-of-words
PV-DM	Distributed Memory version of Paragraph Vector
PV-DBOW	Distributed Bag of Words version of Paragraph Vector
D2V	Doc2Vec
W2V	Word2Vec
DBMS	Database Management System
SQL	Structured Query Language
PSO	Particle Swarm Optimization
RFO	Red Fox Optimization
PBO	Polar Bear Optimization
ChOA	Chimp Optimization Algorithm
